# Whole-Cell MALDI-TOF MS Versus 16S rRNA Gene Analysis for Identification and Dereplication of Recurrent Bacterial Isolates

**DOI:** 10.3389/fmicb.2018.01294

**Published:** 2018-06-19

**Authors:** Michal Strejcek, Tereza Smrhova, Petra Junkova, Ondrej Uhlik

**Affiliations:** Department of Biochemistry and Microbiology, Faculty of Food and Biochemical Technology, University of Chemistry and Technology, Prague, Czechia

**Keywords:** bacterial isolation, bacterial identification, 16S rRNA gene, MALDI-TOF mass spectrometry (MS), MALDI BioTyper, dereplication of isolates, species delineation

## Abstract

Many ecological experiments are based on the extraction and downstream analyses of microorganisms from different environmental samples. Due to its high throughput, cost-effectiveness and rapid performance, Matrix Assisted Laser Desorption/Ionization Mass Spectrometry with Time-of-Flight detector (MALDI-TOF MS), which has been proposed as a promising tool for bacterial identification and classification, could be advantageously used for dereplication of recurrent bacterial isolates. In this study, we compared whole-cell MALDI-TOF MS-based analyses of 49 bacterial cultures to two well-established bacterial identification and classification methods based on nearly complete 16S rRNA gene sequence analyses: a phylotype-based approach, using a closest type strain assignment, and a sequence similarity-based approach involving a 98.65% sequence similarity threshold, which has been found to best delineate bacterial species. Culture classification using reference-based MALDI-TOF MS was comparable to that yielded by phylotype assignment up to the genus level. At the species level, agreement between 16S rRNA gene analysis and MALDI-TOF MS was found to be limited, potentially indicating that spectral reference databases need to be improved. We also evaluated the mass spectral similarity technique for species-level delineation which can be used independently of reference databases. We established optimal mass spectral similarity thresholds which group MALDI-TOF mass spectra of common environmental isolates analogically to phylotype- and sequence similarity-based approaches. When using a mass spectrum similarity approach, we recommend a mass range of 4–10 kDa for analysis, which is populated with stable mass signals and contains the majority of phylotype-determining peaks. We show that a cosine similarity (CS) threshold of 0.79 differentiate mass spectra analogously to 98.65% species-level delineation sequence similarity threshold, with corresponding precision and recall values of 0.70 and 0.73, respectively. When matched to species-level phylotype assignment, an optimal CS threshold of 0.92 was calculated, with associated precision and recall values of 0.83 and 0.64, respectively. Overall, our research indicates that a similarity-based MALDI-TOF MS approach can be routinely used for efficient dereplication of isolates for downstream analyses, with minimal loss of unique organisms. In addition, MALDI-TOF MS analysis has further improvement potential unlike 16S rRNA gene analysis, whose methodological limits have reached a plateau.

## Introduction

Many microbial ecological studies rely on the extraction of bacteria from soil, water, and other environmental samples. In such cases, the number of unique organisms among the hundreds or thousands of isolates is usually limited. It is therefore desirable to separate isolates into bins with common characteristics, i.e., dereplicate them, in order to avoid time-consuming, expensive and, in particular, redundant downstream analyses of each isolate. This dereplication of recurrent bacterial isolates can be achieved by analyzing phenotypic, chemotaxonomic, genotypic, and phylogenetic data (Schleifer, 2009); this can be done by using the following exemplary techniques: fatty acid methyl ester (FAME) profiling of cell membrane lipids or genomic fingerprinting based on repetitive sequence-based polymerase chain reaction—(GTG)_5_-PCR (Versalovic, 1994; Vancanneyti et al., 1996; De Clerck and De Vos, 2002; Coorevits et al., 2008). Small-subunit ribosomal RNA (specifically 16S rRNA) gene sequencing is then often employed to identify and classify representative bacterial isolates (Janda and Abbott, 2007; Kim et al., 2012). The key to the success of 16S rRNA gene sequencing is its applicability across whole bacterial and archaeal domains (Woese, 1987; Munoz et al., 2011). The identification process involves assigning the sequence to a taxonomic bin (phylotype) based on known references either by classification (Wang et al., 2007) or identification of the closest type strain (Kim et al., 2012). Sequence similarity of the 16S rRNA gene can also be used as a proxy for bacterial species; a 98.65% sequence similarity threshold was calculated to best match bacterial species demarcation based on the analysis of 6,787 genomes (Kim et al., 2014).

Ever since its first applications for identification purposes (Claydon et al., 1996; Holland et al., 1996), MALDI-TOF MS has been proposed as a promising alternative for the dereplication of recurrent bacterial isolates (Dieckmann et al., 2005; Ghyselinck et al., 2011; Spitaels et al., 2016) and has been used as a cost- and time-effective alternative to 16S rRNA gene sequencing (Mellmann et al., 2008; Uhlík et al., 2011; Koubek et al., 2012; Wieser et al., 2012; Seng et al., 2013). MALDI-TOF MS-based identification of microorganisms involves the generation of mass spectra from whole-cell material or extracted intracellular content which are then matched to known database references (Fenselau and Demirev, 2001; Lay, 2001). Accurate identification depends on two factors: adequate spectrum quality and close database reference matches. Several successful commercial platforms, such as Biotyper (Bruker Daltonics) and Vitek MS (BioMérieux), are mainly used to identify clinically important species. However, these systems, whose reference databases cover only a small fraction of the vast range of microbial diversity, often fail to function when applied to environmental isolates. Mass spectra generated from whole-cell and cell extract measurements are abundant in ribosomal protein peaks (Ryzhov and Fenselau, 2001; Suarez et al., 2013). Like ribosomal RNA, ribosomal proteins are universally conserved in both prokaryotes and eukaryotes and can be used to reconstruct phylogeny (Yutin et al., 2012).

In this study, we aimed to discuss the current state of reference-based MS classification. In particular, we established mass similarity thresholds which mimic 16S rRNA gene analyses used for species-level delineation based on (i) assigning the closest type strain (herein referred to as phylotype-based approach); and (ii) the 98.65% sequence similarity threshold (herein referred to as sequence similarity-based approach).

## Materials and methods

### Culture collection

The bacterial cultures used in this study consisted of 49 isolates typically found in soils and sediments (Table 1). Twelve strains were previously used as a mock community for error evaluation of high-throughput 16S rRNA gene sequencing analysis (Fraraccio et al., 2017). The other 37 cultures were composed of environmental isolates collected previously by soil/sediment microbial extraction in the authors' laboratory. The bacterial set consisted of three major bacterial phyla (Proteobacteria, Actinobacteria and Firmicutes), spanning five classes, and 22 genera. All cultures were grown on Plate Count Agar (PCA, Oxoid, UK) at 28°C for 24 h.

**Table 1:** Collection of isolates used in this study.

**Culture designation**	**16S rRNA gene analysis Closest type strain (similarity %)**	**MALDI BioTyper™ Identification (Score)**	**Origin**
Rho1	*Rhodococcus erythropolis* NBRC 15567^T^ (99.85)	*Rhodococcus erythropolis* (2.39)	Compost soil
Rho2	***Rhodococcus jostii*** **RHA1** *Rhodococcus jostii* DSM 44719^T^ (99.93)	*Rhodococcus imtechensis* (2.41)	Strain Collection
Rho3	*Rhodococcus pedocola* UC12^T^ (100)	*Rhodococcus* sp. (1.71)	Compost soil
Art1	*Arthrobacter oryzae* NRRL B-24478^T^ (99.42) *Arthrobacter humicola* KV-653^T^ (99.42)	*Arthrobacter* sp. (1.91)	Rhizosphere 1
Art2	*Arthrobacter pascens* DSM 20545^T^ (98.76)	*Arthrobacter oxydans* (2.08)	Rhizosphere 1
Art3	*Arthrobacter halophytocola* KLBMP 5180^T^ (100) *Glutamicibacter arilaitensis* Re117^T^ (100)	*Arthrobacter* sp. (1.87)	Rhizosphere 1
Glu1		*Arthrobacter arilaitensis* (2.56) [*Glutamicibacter arilaitensis*]	Rhizosphere 1
Mic1	***Micrococcus luteus*** **NCTC 2665**^T^	*Micrococcus luteus* (2.53)	Strain collection
Paa1 Paa2 Paa3	*Paenarthrobacter ilicis* DSM 20138^T^ (99.49) *Paenarthrobacter nitroguajacolicus* G2-1^T^ (99.93) *Paenarthrobacter nitroguajacolicus* G2-1^T^ (99.93)	*Arthrobacter ilicis* (2.62) [*Paenarthrobacter ilicisi*] *Arthrobacter aurescens* (2.41) [*Paenarthrobacter aurescens*] *Arthrobacter aurescens* (2.45) [*Paenarthrobacter aurescens*]	Rhizosphere 1 Rhizosphere 1 Rhizosphere 1
Psa1 Psa2	***Pseudarthrobacter chlorophenolicus*** **A6**^T^ *Pseudarthrobacter equi* IMMIB L-1606^T^ (99.93) *Pseudarthrobacter oxydans* KCTC 3383^T^ (99.93)	*Arthrobacter chlorophenolicus* (2.42) [*Pseudarthrobacter chlorophenolicus*] *Arthrobacter chlorophenolicus* (2.12) [*Pseudarthrobacter chlorophenolicus*]	Strain collection Rhizosphere 1
Psa3 Psa4	*Pseudarthrobacter oxydans* KCTC 3383^T^ (100) *Pseudarthrobacter siccitolerans* 4J27^T^ (99.49)	*Arthrobacter oxydans* (2.47) [*Pseudarthrobacter oxydans*] *Arthrobacter polychromogenes* (2.38) [*Pseudarthrobacter polychromogenes*]	Rhizosphere 1 Rhizosphere 1
Oer1	*Oerskovia turbata* NRRL B-8019^T^ (99.85)	*Oerskovia* sp. (1.86)	Rhizosphere 1
Bac1	*Bacillus paralicheniformis* KJ-16^T^ (99.92)	*Bacillus licheniformis* (2.33)	Compost soil
Bac2	*Bacillus rhizosphaerae* SC-N012^T^) (99.5) *Bacillus clausii* DSM 8716^T^ (99.5)	*Bacillus* sp. (1.96)	Compost soil
Bac3	*Bacillus subtilis* subsp. *inaquosorum* KCTC 13429^T^ (100) *Bacillus aryabhattai* B8W22^T^ (100)	*Bacillus megaterium* (2.25)	Compost soil
Bac4	*Bacillus tequilensis* KCTC 13622^T^ (99.93)	*Bacillus subtilis* (2.22)	Compost soil
Bac5 Bac6	*Bacillus safensis* FO-36b^T^ (100) ***Bacillus pumilus*** **SAFR-032** *Bacillus zhangzhouensis* DW5-4^T^ (99.79) *Bacillus pumilus* ATCC 7061^T^ (99.79)	*Bacillus pumilus* (2.08) *Bacillus pumilus* (2.31)	Compost soil Strain collection
Bre1	*Brevibacterium frigoritolerans* DSM 8801^T^ (100) [*Bacillus* sp.]	*Bacillus simplex* (2.2)	Compost soil
Bre2	*Brevibacillus borstelensis* NRRL NRS-818^T^ (99.85)	*Brevibacillus borstelensis* (2.38)	Compost soil
Bre3	*Brevibacillus panacihumi* DCY35^T^ (100)	NA (< 1.7)	Compost soil
Pab1	*Paenibacillus lactis* MB 1871^T^ (99.86)	*Paenibacillus lactis* (2.4)	Compost soil
Lys1	*Lysinibacillus halotolerans* LAM612^T^ (97.86)	*Lysinibacillus* sp. (1.73)	Compost soil
Lys2	*Lysinibacillus halotolerans* LAM612^T^ (99.36)	*Lysinibacillus* sp. (1.72)	Compost soil
Lys3 Lys4	*Lysinibacillus xylanilyticus* DSM 23493^T^ (99.57) *Lysinibacillus xylanilyticus* DSM 23493^T^ (99.22) *Lysinibacillus pakistanensis* JCM 18776^T^ (99.22)	*Lysinibacillus* sp. (1.75) *Lysinibacillus fusiformis* (2.01)	Compost soil Compost soil
Sol1	*Solibacillus isronensis* B3W22^T^ (100)	*Solibacillus silvestris* (2.4)	Compost soil
Spo1	*Sporosarcina koreensis* F73^T^ (99.71)	NA (< 1.7)	Compost soil
Bos1	*Bosea robiniae* DSM 26672^T^ (99.48)	NA (< 1.7)	Rhizosphere 1
Met1	***Methylobacterium radiotolerans*** **JCM 2831**^T^	*Methylobacterium radiotolerans* (2.22)	Strain collection
Rhi1	***Agrobacterium fabrum*** **strain C58** *Rhizobium pusense* LMG 25623^T^ (99.33)	*Rhizobium radiobacter* (2.22)	Strain collection
Ach1	***Achromobacter xylosoxidans*** **A8** *Achromobacter marplatensis* B2^T^ (99.85)	*Achromobacter xylosoxidans* (2.16)	Strain collection
Pan1	***Pandoraea pnomenusa*** **B-356** *Pandoraea pnomenusa* DSM 16536^T^ (99.93)	*Pandoraea pnomenusa* (2.4)	Strain collection
Par1	***Paraburkholderia xenovorans*** **LB400**^T^	*Burkholderia xenovorans* (2.49) [*Paraburkholderia xenovorans*]	Strain collection
Cup1	***Cupriavidus necator*** **H850** *Cupriavidus necator* N-1^T^ (99.93)	*Cupriavidus necator (2.36)*	Strain collection
Psm1 Psm2	***Pseudomonas alcaliphila*** **JCM 10630**^T^ ***Pseudomonas alcaliphila*** **JAB1** *Pseudomonas chengduensis* MBR^T^ (99.93)	*Pseudomonas alcaliphila* (2.41) *Pseudomonas alcaliphila* (2.24)	Strain collection Strain collection
Psm3	*Pseudomonas anguilliseptica* NCIMB 1949^T^ (99.33)	*Pseudomonas anguilliseptica* (2.02)	Rhizosphere 1
Psm4 Psm5	*Pseudomonas extremaustralis* 14-3^T^ (99.86) *Pseudomonas gessardii* DSM 17152^T^ (99.93)	*Pseudomonas veronii* (2.37) *Pseudomonas gessardii* (2.25)	Sediment 1 Sediment 1
Psm6 Psm7 Psm8 Psm9	*Pseudomonas hunanensis* LV^T^ (99.64) ***Pseudomonas putida*** **JB** *Pseudomonas hunanensis* LV^T^ (99.93) *Pseudomonas hunanensis* LV^T^ (99.93) *Pseudomonas taiwanensis* BCRC 17751^T^ (98.92)	*Pseudomonas* sp[2] (2.31) *Pseudomonas putida* (2.42) *Pseudomonas putida* (2.45) *Pseudomonas* sp. (1.79)	Contaminated soil Rhizosphere 2 Sediment 2 Sediment 1
	*Pseudomonas plecoglossicida* NBRC 103162^T^ (98.92)		
Psm10	***Pseudomonas stutzeri*** **JM300** *Pseudomonas songnenensis* NEAU-ST5-5^T^ (99.06)	NA (< 1.7)	Strain collection

*Identification results based on 16S rRNA gene (EzBioCloud Identify Service) and MALDI BioTyper (v3.1 equipped with MBT 6903, covering 2,226 unique bacterial species) analyses. Entries in bold are known bacterial strains. Cultures in rectangles were grouped by 98.65% similarity of the 16S rRNA gene using the UPGMA clustering method. Entries in square brackets are updated bacterial nomenclature entries (see Materials and Methods section). Origin: compost soil—compost soil for gardening purposes, Central Bohemia, Czech Republic; rhizophere 1—PCB-contaminated soil with horseradish vegetation, South Bohemia, Czech Republic (Uhlík et al., 2011); rhizosphere 2—PCB-contaminated soil with nightshade vegetation, Northern Bohemia, Czech Republic (Kurzawová et al., 2012); contaminated soil—PCB-contaminated soil, Czech Republic (Nováková et al., 2002); sediment 1—PAH-contaminated sediment, Romania (Wald et al., 2015); sediment 2—PCB-contaminated sediment, Strazsky kanal, Slovakia (Koubek et al., 2012). All sequences of environmental isolates were deposited in the NCBI Nucleotide database under PopSet number 1315444717. Accession numbers for the strains obtained from microbial collections are as follows: Achromobacter xylosoxidans A8 (NC_014640); Agrobacterium fabrum strain C58 (NC_003062); Bacillus pumilus SAFR-032 (NC_009848); Cupriavidus necator H850 (MG708169); Methylobacterium radiotolerans JCM 2831^T^ (NC_010505); Micrococcus luteus NCTC 2665^T^ (NC_012803); Pandoraea pnomenusa B-356 (EF596910); Paraburkholderia xenovorans LB400^T^ (NC_007951); Pseudarthrobacter chlorophenolicus A6^T^ (NC_011886); Pseudomonas alcaliphila JAB1 (NZ_CP016162); Pseudomonas alcaliphila JCM 10630^T^ (NR_024734); Pseudomonas putida JB (NZ_CP016212); Pseudomonas stutzeri JM300 (MG708165) and Rhodococcus jostii RHA1 (NC_008268)*.

### DNA isolation and 16S rRNA gene PCR amplification

Genomic DNA was isolated from pure cultures using thermal lysis. Briefly, an entire loop of cell material was resuspended in molecular grade water (Sigma-Aldrich, USA) and incubated at 99°C for 15 min. The lysates were pelleted, and the supernatant was used as a template DNA source. The PCR mixture, with a total volume of 15 μL, was prepared using the KAPA HiFi HotStart ReadyMix kit (Kapa Biosystems, USA) and 16S rRNA gene primers 27fM, 5′-AGAGTTTGATCMTGGCTCAG-3′ and 1492rY, 5′-GYTACCTTGTTACGACTT-3′ (Lane, 1991). The PCR thermal profile was set to 95°C for 5 min, followed by 25 cycles of 98°C for 20 s, 56°C for 15 s, and 72°C for 45 s, and concluded with a final elongation step at 72°C for 5 min. After the PCR products were evaluated by 1% agarose gel electrophoresis, 3–6 additional cycles of reconditioning PCR (Thompson et al., 2002) were performed with 5 μL of PCR product as a template DNA to obtain a final volume of 50 μL. Samples were purified with the Genomic DNA Clean & Concentrator™-10 Kit (Zymo Research, USA) following the manufacturer's instructions. Sanger sequencing was performed bidirectionally using both forward and reverse primers at GATC BIOTECH, Konstanz, Germany. Sanger sequencing chromatograms were manually inspected with the aid of MEGA7 software (Kumar et al., 2016), converted into sequences and both reads were then merged into a nearly full-length sequence. All sequences were trimmed to the corresponding *Escherichia coli* 16S rRNA gene positions 57 to 1449 and were deposited in the NCBI nucleotide database under PopSet number 1315444717.

### 16S rRNA gene sequence analysis

Almost full-length 16S rRNA gene sequences were uploaded to EzBioCloud (Yoon et al., 2017) and classified using the Identify service (Version 2017.05). The closest type strain match was used for potential species identification (Kim et al., 2014). Sequences sharing the assigned closest type strain are herein designated as species-level phylotypes and are, for simplicity, referred to as phylotypes throughout this study. All multiple type strains with the same percent similarity to the culture tested were reported (Table 1).

As a complementary approach to grouping closely related bacterial cultures without reliance on referential databases, a similarity-based clustering was employed. Sequence pairwise similarities of 16S rRNA genes were obtained by creating global pairwise alignments (Needleman and Wunsch, 1970) and calculating their percent sequence identity using the Bioconductor R package (Huber et al., 2015). In accordance with the techniques outlined by Kim et al. (2014), the internal gap positions were not included in the similarity calculations. Operational taxonomic units were constructed using UPGMA cluster analysis, with a distance cutoff of 98.65% sequence similarity, which was previously reported as the closest proxy of species (Kim et al., 2014) and were further labeled as OTUs_[98.65%]_.

### MALDI-TOF MS sample preparation and spectra acquisition

Prior to MALDI-TOF MS measurement, bacterial isolates were freshly inoculated on PCA (Oxoid, UK) and cultivated for 24 h at 28°C. The common direct transfer protocol (commonly referred to as whole-cell or intact-cell measurement) was followed to obtain mass spectra. Briefly, ~0.1 mg of cell material was directly transferred from a bacterial colony (if possible) or smear of colonies to a MALDI target spot. After drying at laboratory temperature, sample spots were overlaid with 1 μL of matrix solution (10 mg/mL α-cyano-4-hydroxycinnamic acid in 50% acetonitrile and 2.5% trifluoroacetic acid). To determine mass spectra generation reproducibility, all cultures were cultivated independently four times (biological replicates); each measurement was carried out in triplicate (technical replicates). MS analysis was performed on an Autoflex MALDI-TOF mass spectrometer (Bruker Daltonics, Germany) using Flex Control 3.4 software (Bruker Daltonics, Germany). Calibration was carried out with the use of the Bacterial Test Standard (Bruker Daltonics, Germany).

All MS spectra were measured automatically using Flex Control software according to the standard measurement method for microbial identification. Specifically, our set-up values in linear positive mode were as follows: ion source 1 voltage, 20 kV; ion source 2 voltage, 19 kV; lens voltage, 6.5 kV; mass range, 2–20 kDa; the final spectrum was the sum of 10 single spectra, each obtained by 200 laser shots on random target spot positions. With regard to the functioning of MALDI-TOF MS, by which +1 ions are predominantly generated and detected, Da is used as a unit of *m*/*z* throughout the study.

### Bruker biotyper bacterial classification and identification

For bacterial classification using BioTyper 3.1 software (Bruker Daltonics, Germany) equipped with MBT 6903 MPS Library (released in April 2016), the MALDI Biotyper Preprocessing Standard Method and the MALDI Biotyper MSP Identification Standard Method adjusted by the manufacturer (Bruker Daltonics, Germany) were used. All identifications were reported with the following score values: < 1.7 was interpreted as an unreliable identification; 1.7–2.0 as a probable genus identification; 2.0–2.3 as a secure genus identification and probable species identification; and >2.3 was regarded as a highly probable species identification. Only the highest score value of all mass spectra belonging to individual cultures (biological and technical replicates) was recorded. Mismatched identifications between MALDI BioTyper and 16S rRNA gene analyses, which could be resolved by recent nomenclature changes in the EzBioCloud database, as well as the special case of culture Bre1, were not regarded as misidentifications. Nomenclature changes included genera *Arthrobacter* (Busse, 2016), *Burkholderia* (Sawana et al., 2014), and *Agrobacterium* (Lassalle et al., 2011). Culture Bre1, which showed 100% 16S rRNA gene sequence similarity to type strain *Brevibacterium frigoritolerans* DSM 8801^T^ using the EzBioCloud Identify service, was, however, identified as *Bacillus simplex* using the MALDI BioTyper method (score of 2.2). Further inspection carried out in-house by DSMZ culture collection, based on multiple taxonomic tests including DNA-DNA hybridization experiments, revealed that the strain DSM 8801^T^ is actually a member of *Bacillus* sp. (personal communication).

### Mass spectra preprocessing

All MS data were processed in R language (R. Core Team, 2017) with the aid of the *MALDIquant* R package (Gibb and Strimmer, 2012). The workflow followed standard spectral data preprocessing procedures adopted from the *MALDIquant* package: (i) square root intensity transformation; (ii) mass range trimming of 4–10 kDa (see results for details); (iii) Savicky-Golay intensity smoothing (Savitzky and Golay, 1964) with a half-window size of 20; (iv) baseline correction of spectra by the SNIP algorithm (Morhac, 2009) with 50 iterations; (v) total ion current normalization; (vi) peak detection using the SuperSmoother noise estimation algorithm (Friedman, 1984), with a signal-to-noise ratio of 3 and a half-window size set to 20; and (vii) peak binning with 0.002 tolerance.

Peak lists of individual spectra were transformed into a feature matrix with mass signal positions marked in columns. In cases where spectra were lacking specific peaks, the corresponding intensity values of preprocessed spectra was used. Spectral pairwise similarities were calculated as cosine similarities (Stein and Scott, 1994). For fast and efficient computing, the *cosine()* function implemented in the *coop* R package (Schmidt, 2016) was used. If required, distance was calculated using the formula 1 – the CS.

#### MALDI-TOF MS reproducibility assessment

**Figure 1:**
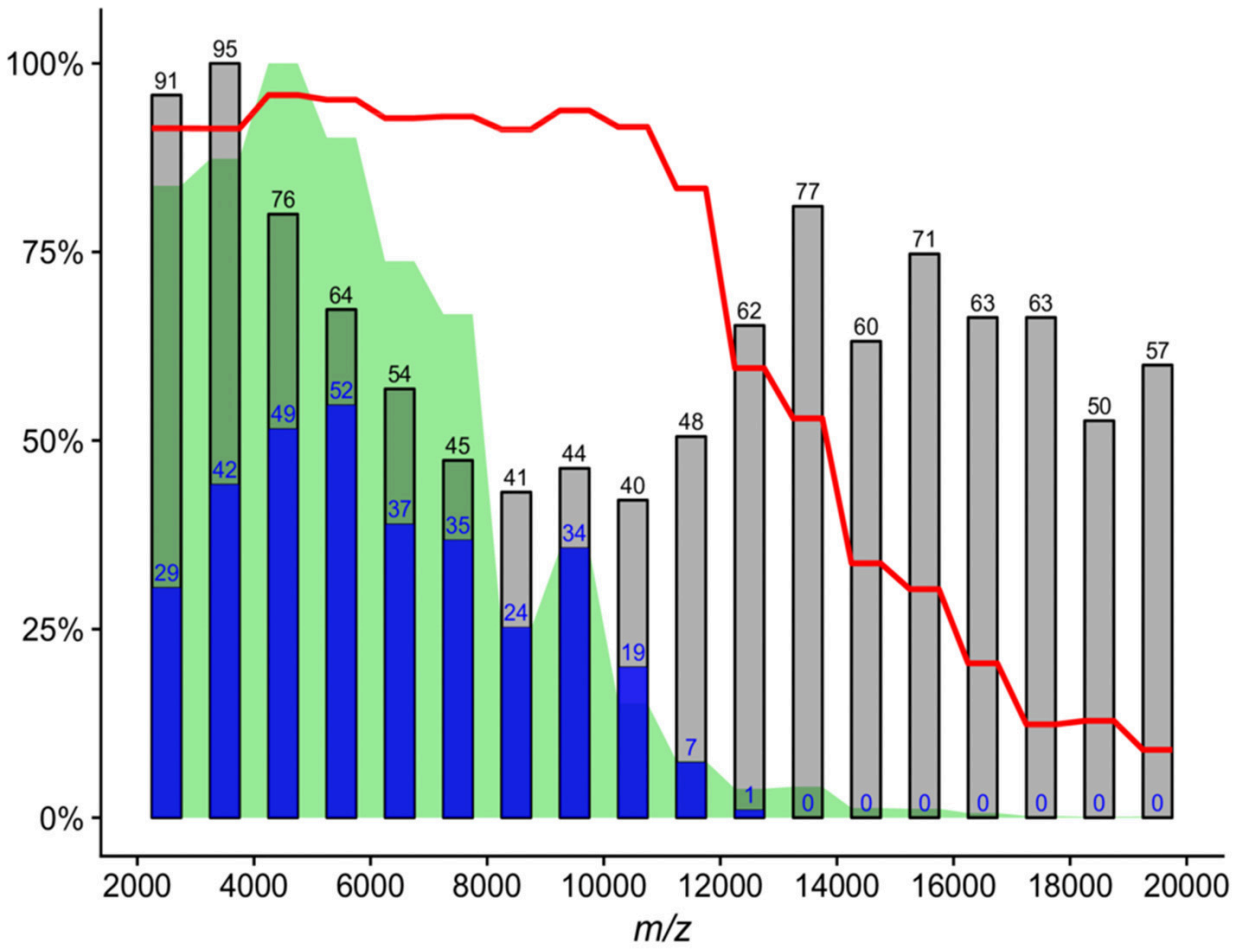
Analysis of 1 kDa mass intervals across all 585 mass spectra. *Gray bars*—number of detected mass signals per interval; *blue bars*—number of mass signals identified by shrinkage discriminant analysis as useful for species prediction based on assigning the closest type strain; *green area*—proportional mass signal intensity; *red line*—mean value of average cosine similarity between biological and technical replicates of individual cultures (4 × 3 = 12 spectra). All values are normalized by maxima of the respective variable.

Prior to analysis, low quality spectra were identified by calculating the average cosine similarity (ACS) between each spectrum and its corresponding technical replicates. A 0.9 cutoff was derived from the shape of the distribution of these values to determine technical outliers. Out of the mass spectra totaling 588, three were discarded. The reproducibility of the MS measurements was evaluated by calculating ACS in groups of technical and biological replicates. In addition, the full 2 to 20 kDa mass range was split into 1 kDa intervals, for each of which we calculated: (i) the number of unique mass signals; (ii) the summed signal intensity; and (iii) the mean of the ACS values calculated for all mass spectra belonging to each individual culture (12 spectra; Figure 1).

### Identification of phylotype-predicting mass signals

To identify species-level phylotype-predicting mass signals, shrinkage discriminant analysis with correlation-adjusted *t*-score variable selection (Ahdesmaki and Strimmer, 2010) as implemented in the *sda* R package (Ahdesmaki et al., 2015) was carried out. The signals were detected in the whole 2 to 20 kDa mass range. All peaks were ranked on a mutual information entropy basis, and selection was controlled by the false non-discovery rate. All peaks with a local false discovery rate of less than 0.2 were selected as phylotype predictors. Prediction accuracy was estimated using 10 × 10-fold cross validation of all MS data with the aid of the *crossval* R package (Strimmer, 2015) as described in *sda* documentation.

### Optimal cosine similarity threshold for species-like separation based on 16S rRNA gene analysis

Cosine similarity (CS) was chosen as a measure of similarity between mass spectra. Geometrically, it is interpreted as a cosine of the angle between two vectorized mass spectra. It is calculated as a normalized inner product, with CS values ranging between 0 and 1, as mass intensities are always positive.

A dataset containing all MS measurement pairs was constructed, and spectral CS was computed for each pair. If a sample-sample pair was assigned to the same closest type strain, the pair was labeled as *intra*-related; otherwise, it was labeled as *inter*-related. To determine the optimal threshold for mass spectra cosine similarity (*T*_*CS*_), the precision, recall and F_1_ scores were calculated for each CS threshold value (0–1 with 0.01 steps) and evaluated with 2-fold cross validation as described by Kim et al. (2014). All sample-sample pairs were tested for species-like relatedness and were designated as: True Positive (*TP*) if CS > = *T*_*CS*_ & *intra*; True Negative (*TN*) if CS < *T*_*CS*_ & *inter*; False Positive (*FP*) if CS > = *T*_*CS*_ & *inter*; and False Negative (*FN*) in CS < *T*_*CS*_ & *intra*. The dataset was then randomly split into two partitions, and the precision [*TP / (TP* + *FP)*], recall [*TP / (TP* + *FN)*] and F_1_ scores [*2* × *(precision* × *recall) / (precision* + *recall)*] were calculated for each CS threshold value in relation to each partition. Optimal *T*_*CS*_ was selected as the mean of the thresholds with the highest F_1_ score from both cross validation training partitions. The precision and recall scores of the thresholds selected were calculated on the basis of the corresponding test partition. Similarly, the whole procedure was performed for species delineation using the sequence similarity approach. When sample-sample pairs shared 16S rRNA gene sequence similarity ≥98.65%, the pair was labeled as *intra*-related; otherwise, it was labeled as *inter*-related.

Operational taxonomic units were constructed using UPGMA cluster analysis on MS data with specified CS threshold and were herein labeled as OTUs_[CS threshold]_.

### Bacterial ribosomal protein molecular weights

UniProtKB protein database (UniProt Consortium, 2017) was searched for “taxonomy:bacteria family:ribosomal” protein entries. In total, 761,208 proteins were found including entries from both reviewed (Swiss-Prot) and unreviewed (TrEMBL) sources, and their calculated molecular masses were downloaded. No post-transcriptional or other modifications were applied in the mass calculations.

### R data analysis scripts deposition

All scripts used for analyses in R are available at the authors' GitHub repository (https://github.com/strejcem/MALDIvs16S/).

## Results

### Classification of cultures based on 16S rRNA gene and MALDI-TOF MS reference databases

With the aid of the EzBioCloud Identify service, the culture set was found to consist of 43 phylotypes (Table 1). Bruker MALDI BioTyper software with a reference database was used to identify and classify the cultures according to their mass spectra (Table 1). Of the 49 cultures studied, 45 were reliably identified at the probable genus level, with BioTyper scores of >1.7. After taking into account recent taxonomy changes and corrections described in the Materials and Methods section, the MALDI BioTyper and phylotype-based identification methods coincided up to the genus level. With respect to only those cases where MALDI BioTyper identifications reached scores of >2.3 (highly probable species identification; 23 cultures), 12 cultures were assigned to the same species as by the phylotype-based approach. Lowering the score cutoff to 2.0 (secure genus identification and probable species identifications; 36 cultures) resulted in 15 concordant species assignments. With regard to all 49 cultures, both identification methods yielded the same overall genus and species assignments in 92 and 35% of cases, respectively.

### Similarity-based analysis of whole-cell mass spectra: mass range determination

The entire set of mass spectra was transformed into a feature matrix and the number of descriptive statistics was calculated for each 1 kDa interval in the full 2–20 kDa mass window (Figure 1). It is important to note that up to 94% of summed signal intensities were in the 2–10 kDa range. The mean of ACS values, which were highly consistent (ACS > 0.9) up to 11 kDa followed by a rapid deterioration, showed a similar trend. Shrinkage discriminant analysis was also performed to identify specific protein signals for species assignment using the phylotype-based approach (Figure 1). Out of 1,101 unique protein signals, 150 were found to be adequate for phylotype prediction. Prediction accuracy, calculated by cross validation, was 0.999, meaning that on average less than one out of 585 cases was incorrectly predicted. The ratio between phylotype-specific and total mass signals increased significantly in the 4–10 kDa range (Figure 1).

**Figure 2:**
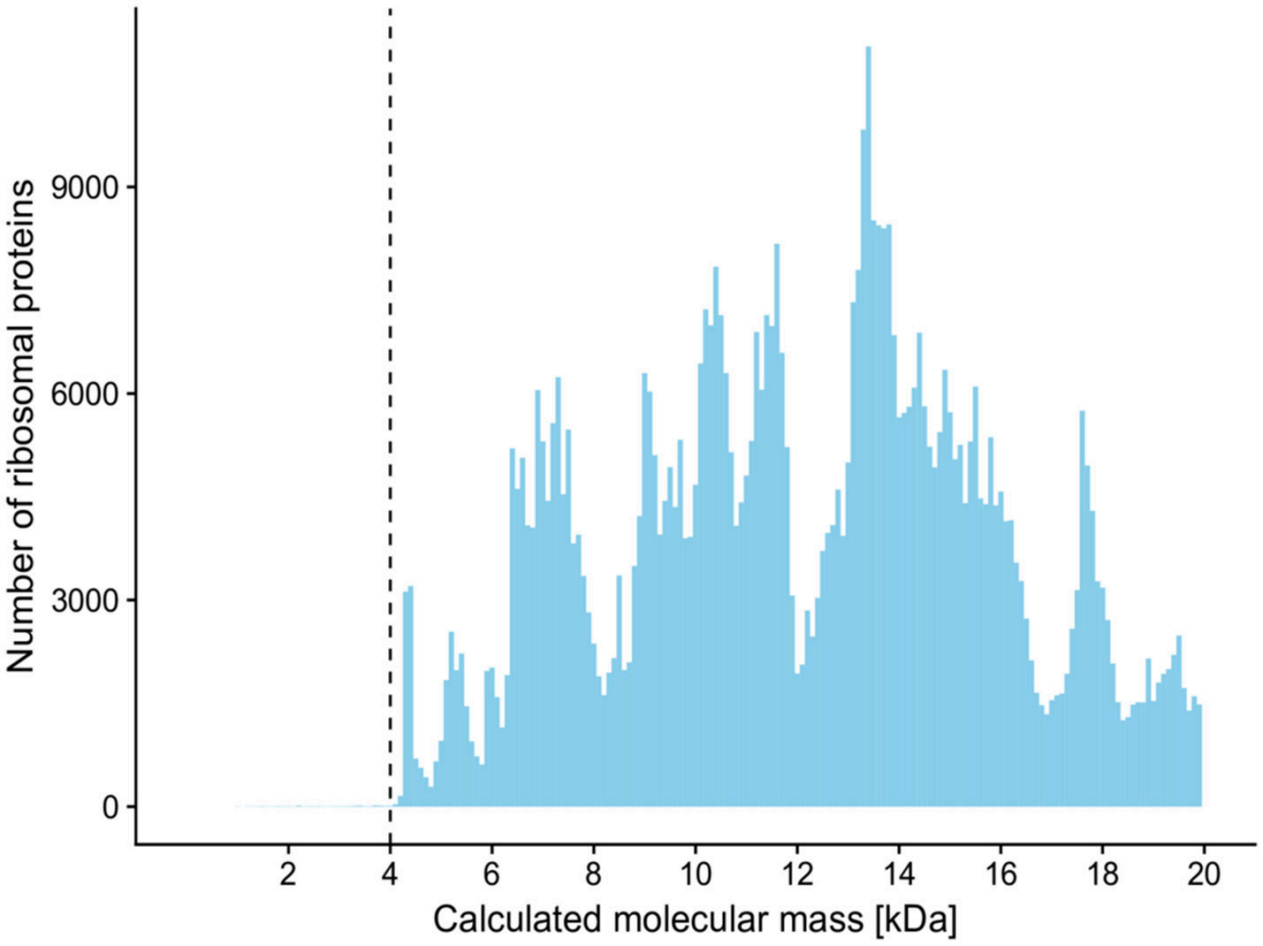
Histogram of calculated molecular masses of bacterial ribosomal proteins. Only 123 (0.01%) proteins out of 761,208 had a molecular mass of less than 4 kDa. Molecular masses were calculated and downloaded from the UniProtKB protein database. The histogram is made up of bins of 100 Da, and only proteins with a mass of less than 20 kDa are shown.

Although the 10–20 kDa mass range was characterized by many mass signals, their summed intensity was 6% of full 2–20 kDa range signal intensity. Protein signals in the 2–4 kDa mass range accounted for 29% of full 2–20 kDa range signal intensity. Overall, the 2–4 kDa mass range contained 186 unique mass signals across all spectra, with an average of 12.2 mass signals per spectrum; however, only 17 (9%) unique signals had phylotype-discriminating capacity. By comparison, the mass range of 4–10 kDa accounted for 65% of all intensities, with an average of 22.9 mass signals per spectrum. Out of 324 unique peaks localized in this MS range, 127 (39%) were found to have phylotype-discriminating capacity. Analysis of 761,208 bacterial ribosomal proteins downloaded from the UniProtKB protein database also showed that only 123 (0.01%) proteins had a calculated molecular mass of less than 4 kDa (Figure 2). In light of these findings, a mass range of 4–10 kDa was used for the analyses described below in order to reduce data complexity and signal noise. Further evaluation of ACS per culture using the full 2–20 kDa range as opposed to the restricted 4–10 kDa range indicated that the mass range restriction had a largely positive impact (Supplementary Figure 1).

### Reproducibility of mass spectra

**Figure 3:**
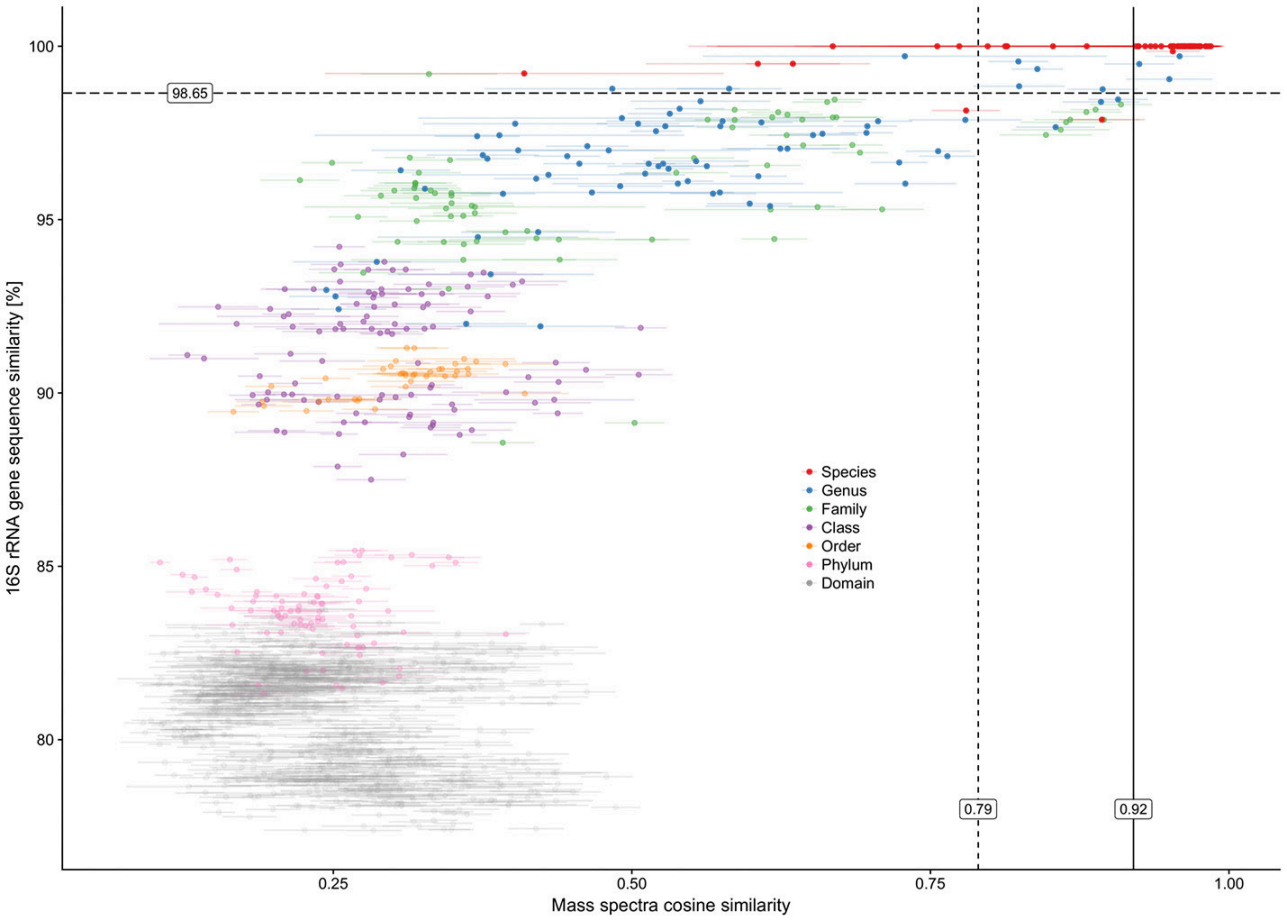
Plot showing pairwise relationship of 16S rRNA gene sequence similarity between two cultures and their mass spectra cosine similarity. Horizontal error bars represent standard deviations of mass spectra cosine similarities calculated for all technical and biological replications (*n* = 3 × 4 = 12). The colors of the data points represent the lowest taxonomy rank that is shared between a pair of microorganisms. The taxonomical classification and species assignment by the closest type strain were carried out using the EzCloud Identify Service. *Vertical solid line*—calculated optimal cosine similarity threshold based on 98.65% 16S rRNA gene similarity; *vertical dashed line*—calculated optimal cosine similarity threshold based on assigning the closest type strain.

The ACS calculated between technical replicates varied from 0.916 to 0.997, thus indicating a high level of overall mass spectra reproducibility when the same cell material was analyzed. However, the ACS calculated over all 12 spectra belonging to each individual culture revealed significant misalignment between a certain number of biological replicates. ACS values for the biological replicates of all 49 cultures were in a 0.756 to 0.985 range, with eight cultures showing an ACS of less than 0.9. These cultures (mean ACS ± std. dev.) included: Bac1 (0.813 ± 0.162), Bac2 (0.800 ± 0.107), Bac3 (0.814 ± 0.143), Bac4 (0.756 ± 0.193), Bac5 (0.814 ± 0.178), and Lys3 (0.774 ± 0.203) belonging to bacterial class Bacilli (6 out of 16); Met1 (0.881 ± 0.066) belonging to class Alphaproteobacteria (1 out of 3); and Psm7 (0.853 ± 0.138) belonging to class Gammaproteobacteria (1 out of 10). No misalignment of biological replicates between individual cultures was detected with respect to Actinobacteria (0 out of 16) or Betaproteobacteria (0 out of 4). On the whole, Gram-negative cultures were found to be less affected than Gram-positives. No linear dependency was observed between mass spectra CS and 16S rRNA gene sequence similarities (Figure 3). All mass spectra pairs with a spectral similarity of over 0.60 coincided in terms of family and deeper taxonomic ranks, while pairwise mass spectra similarity of cultures of the same species-level phylotype ranged from as low as 0.232 to 0.998 (Figure 3).

### Optimal CS threshold to delineate species analogically to the phylotype-based approach

**Figure 4:**
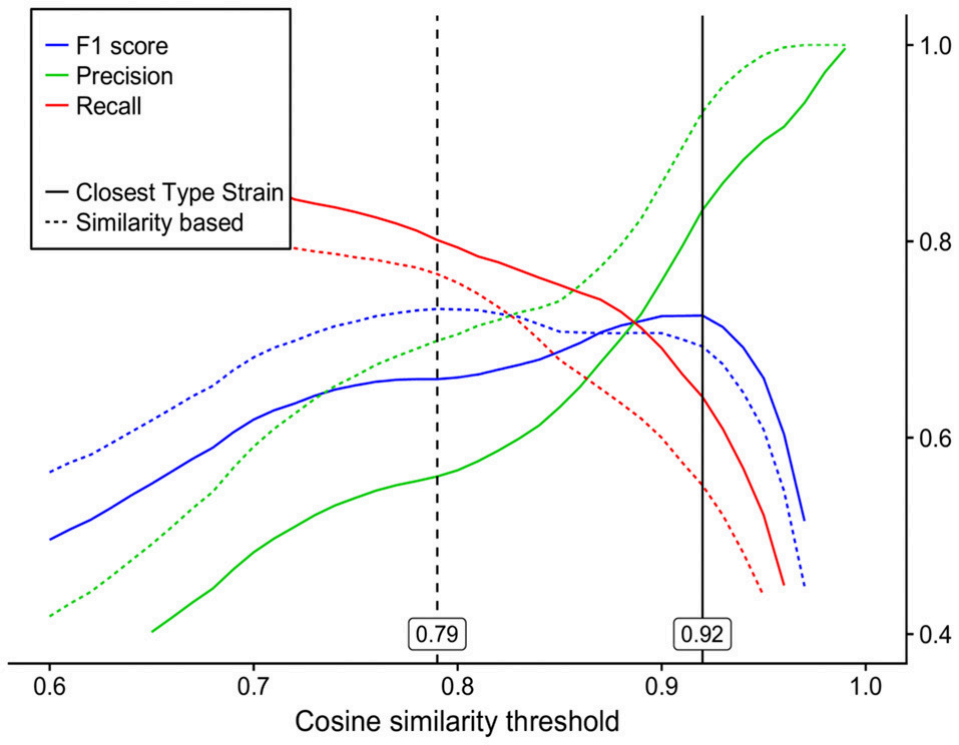
Precision, recall and F_1_ score curves for species classification by mass spectra cosine similarity (*x*-axis) as compared to the two commonly used 16S rRNA gene species demarcation analyses: *dashed lines*—species separation by 98.65% 16S rRNA gene similarity with an optimal analogous cosine similarity threshold of 0.79; *solid lines*—species assignment by the closest type strain (EzCloud Identify Service) with an optimal analogous cosine similarity threshold of 0.92.

Using 2-fold cross validation, the CS threshold calculated on an F_1_ score basis was 0.92 and differentiated mass spectra analogously to the phylotype-based approach. The corresponding precision and recall values were 0.83 and 0.64 (Figure 4).

Altogether, the 49 different cultures in four biological replicates used in this study represented a mass spectra dataset of 196 biological samples. Using 16S rRNA gene analysis, the collection was found to be composed of 45 unique phylotypes. UPGMA cluster analysis with a CS threshold of 0.92 resulted in the generation of 76 clusters (OTUs_[CS0.92]_). Of these, 32 OTUs_[CS0.92]_ were actually duplicated (redundant) due to the biological variability of the mass spectra. While leaving out redundant clusters, 39 out of 49 cultures were separated analogically using both methods, five cultures were separated into more phylotypes than OTUs_[CS0.92]_ and, finally, five cultures were separated into more OTUs_[CS0.92]_ than phylotypes (Table 2).

**Table 2:** Comparison of MALDI-TOF MS and 16S rRNA gene analysis methods for dereplication of recurrent bacterial isolates.

**Cosine Similarity threshold**	**16S rRNA gene analysis**	**Number of clusters MS/rRNA**	**Dereplication rate MS/rRNA (% of samples)**	**Redundant MS clusters (% of clusters)**	Cultures separated by^*^:		
					**Both approaches^a^**	**rRNA^b^**	**MS^c^**
0.79	98.65% similarity	46/37	23%/19%	8 (17%)	35	6	8
0.92	Closest type strain	76/43	39%/22%	32 (42%)	39	5	5

*Bacterial set samples are represented by 4 biological replications of 49 cultures (196 samples in total)*.

* *Number of cultures that were (a) separated in the same way by both MALDI-TOF MS and 16S rRNA gene analysis, (b) separated into more clusters by 16S rRNA gene analysis and (c) separated into more clusters by MALDI-TOF MS*.

### Optimal CS threshold to delineate species analogically to the sequence similarity-based approach

The optimal CS threshold corresponding to the 98.65% sequence similarity threshold (Kim et al., 2014) was calculated as 0.79, with precision and recall values of 0.70 and 0.73, respectively (Figure 4). UPGMA cluster analysis resulted in the generation of 37 OTUs_[98.65%]_ using 16S rRNA gene data and of 46 OTUs_[CS0.79]_ using MS data, 8 of which were redundant. Leaving out redundant OTUs_[CS0.79]_, 35 out of 49 cultures were separated in the same way by both methods, 6 cultures were grouped into more OTUs_[98.65%]_ than OTUs_[CS0.79]_ and 8 cultures were separated into more OTUs_[CS0.79]_ than OTUs_[98.65%]_ (Table 2). Supplementary Figure 2 shows a mass spectrum UPGMA dendrogram, with clusters marked for each cutoff. In summary, the MALDI-TOF MS technique, when used to dereplicate bacterial isolates, leads to a reduction in the number of isolates for downstream analyses, with minimal loss of unique organisms.

## Discussion

### Bacterial identification based on reference databases

MALDI-TOF mass spectrometry is now well-established as a fast and reliable technique in clinical laboratories to identify bacterial species (Janda and Abbott, 2007; Croxatto et al., 2012; Seng et al., 2013; Shin et al., 2015; Buckwalter et al., 2016), although its application in the field of microbial ecology has been more limited. In this study, we compared whole-cell MALDI-TOF MS analysis of environmental isolates to the standard 16S rRNA gene sequencing method for identification and characterization of bacteria.

In environmental studies, fast and effective bacterial classification is principally based on matching sample 16S rRNA gene sequences to known references in databases such as the SILVA rRNA database project (Quast et al., 2013), whose latest release 128 consists of 1,922,213 high-quality full-length 16S rRNA sequences, and the Ribosomal Database Project (Cole et al., 2014), whose release 11.5 contains 1,502,575 high quality, aligned and annotated 16S rRNA sequences. The manually curated 16S rRNA gene database EzBioCloud, which is used for the closest type strain bacterial identification (Kim et al., 2012), contains almost 60,000 bacterial type strains and uncultivated phylotypes in version 2017.05. By comparison, the latest MALDI BioTyper library, released in April 2016, contains 6,127 reference mass spectra (main spectrum projections, MSP) with 2,226 unique bacterial species. Both the EzBioCloud Identify service and the MALDI Biotyper database closely coincided in terms of identifying the cultures examined at the genus level, with all cultures matching, except for 4 unreliably identified by the MALDI BioTyper method. Reference-based MALDI-TOF MS classification thus proved to be a reliable technique for bacterial identification at the genus level provided a wide coverage of reference mass spectra is available. However, at the species level, only 15 (~35%) of the cultures identified coincided with those identified by 16S rRNA gene sequencing analysis. These findings are in contrast to previous studies of clinically important bacteria where concordant species identifications between MALDI BioTyper and 16S rRNA gene analysis were reported to be in the range 41–92.2% of samples (Mellmann et al., 2008; Bizzini et al., 2011; Schmitt et al., 2013; Cheng et al., 2015; Fykse et al., 2015; Schulthess et al., 2016). This discrepancy is most likely due to the insufficient coverage of bacterial species in the databases. Even though users of commercial databases can create local repositories (Schmitt et al., 2013; Cheng et al., 2015; Svobodova et al., 2017), there is no centralized pooling of new references collected by a broad spectrum of researchers. Although open access microbial MS databases such as SpectraBank (Bohme et al., 2012) and Spectra (spectra.folkhalsomyndigheten.se/) have existed for many years, growth in the number of uploaded spectra has been slow or stagnant. The lack of widely accepted guidelines on the production of MALDI-TOF mass spectra (Liu et al., 2007) or on the data format to be adopted—SpectraBank, with plain text peak lists without intensities or SpectraBank, with its Bruker MSP proprietary format—may hinder further progress in the adoption of the MALDI-TOF MS method for bacterial classification and identification, especially in environmental studies.

### Mass spectra preprocessing

One of the main goals of our study was to provide parameters which could result in the efficient use of the MALDI-TOF MS without reliance on mass spectra reference databases for the dereplication of recurrent bacterial isolates from environmental samples in such a way it would be analogical to 16S rRNA gene-based analyses. We attempted to identify mass range with stable and predictive protein signals prior to CS calculation, which resulted in the selection of a mass range of 4–10 kDa. Mass signals in the 10–20 kDa range were unlikely to be reproducible protein peaks, as shown by the decreasing ACS values between spectra assigned to the same culture (Figure 1). Although the largest number of mass signals were in the mass range of 2–4 kDa, the frequency of phylotype-predictive signals detected by shrinkage discriminant analysis, was low, suggesting that incorporation of this region into the calculation of similarity measures is not essential. Several other studies suggest that the 3.5–4 kDa mass range is the lower boundary where important signals are located (Arnold and Reilly, 1998; Fenselau and Demirev, 2001). Dieckmann et al. (2005) only considered high intensity and stable signals with a mass of less than 4 kDa. These findings are further corroborated by analysis of bacterial ribosomal proteins extracted from the UniProtKB protein database which showed a very limited number of such proteins with a molecular mass of under 4 kDa (Figure 2).

### Cosine similarity thresholds vs. 16S rRNA gene analysis

Optimal CS thresholds delineating cultures analogously to both phylotype- and sequence similarity-based 16S rRNA gene analysis approaches were identified based on the F_1_ score which is defined as the harmonic mean of precision and recall values. Within the scope of this study, following dereplication by MALDI-TOF MS, high precision values would translate into a slight loss of unique phylotypes/OTUs_[98.65%]_ identified by the 16S rRNA gene analysis, while high recall values would translate into a limited number of redundant clusters of the same phylotype/OTUs_[98.65%]_.

On the basis of a 2-fold cross validation and F_1_ score calculation, optimal CS thresholds of 0.79 and 0.92 were identified to best mimic species separation defined by the phylotype-based and sequence similarity-based approaches, respectively. The precision values for the respective thresholds were 0.70 and 0.84. In order to dereplicate recurrent bacterial isolates, a further increase in the CS threshold might yield a higher level of precision, although this would be at the expense of a lower recall rate (Figure 4). The recall values for the CS thresholds of 0.79 and 0.92 were 0.77 and 0.55, respectively. These values would, on average, imply 23 and 45% redundant clusters, respectively, upon dereplication. These recall values might be negatively influenced by two major factors: biological reproducibility (see below) and the fact, that these values relate to 16S rRNA gene analysis which is used only *as a proxy* for bacterial species delineation and should be applied with caution. The overall conserved character of the 16S rRNA gene, making it applicable to virtually all prokaryotic organisms, does not allow for subspecies separation and, in some cases, not even for species separation (Fox et al., 1992). Prokaryotic species are nowadays defined using whole-genome-based techniques, such as average nucleotide identity (ANI) or DNA-DNA hybridization (Stackebrandt and Goebel, 1994; Konstantinidis et al., 2006; Tindall et al., 2010). Kim et al. (2014) have reported precision and recall values of 0.922 and 0.986, respectively, when a 98.65% 16S rRNA gene sequence similarity threshold was used to delineate species defined by 95% ANI. If the actual species-defining approaches were used as reference methods, the recall values for MALDI-TOF MS analysis would very likely increase. In this study, cultures Lys3 and Lys4 of single phylotype *Lysinibacillus xylanilyticus* shared 99.2% similarity of 16S rRNA gene sequences, while their ACS was 0.410 ± 0.167. Similarly, *Arthrobacter* Art3 and *Glutamicibacter* Glu1 shared 99.2% sequence similarity between their 16S rRNA genes, while their ACS was 0.330 ± 0.057. This strongly suggests that the resolution of the MALDI-TOF MS technique is superior to that of 16S rRNA gene analysis in particular cases, as was also described elsewhere (Murray, 2010; Böhme et al., 2013). Taking all this into account, further in-depth research into cultures with known genomes is required in order to provide more robust similarity threshold values for species demarcation by MALDI-TOF MS.

### Effect of biological variation

While the technical replicates of MALDI-TOF MS measurement showed a high level of reproducibility, the biological replicates of some culture mass spectra deviated significantly. These deviations distorted the F_1_ score curves (Figure 4) and artificially lowered the CS threshold calculated for species delineation. Enhanced precision and recall could be expected if a higher level of biological reproducibility was achieved. Oberle et al. (2016), after studying the technical, biological and interlaboratory reproducibility yielded by MALDI-TOF MS cell analysis, came to conclusions which are in line with our findings. Using 12 *E*. *coli* strains and standard operating procedures, they reported satisfactory technical, but insufficient biological reproducibility with regard to similarity-based analyses. Despite this low level of biological reproducibility, they were able to identify cluster-determining peaks which facilitated accurate classification of all samples. Using shrinkage discriminant analysis, we were able to identify 150 phylotype-specific mass signals in our dataset. Using these 150 mass signals, it was possible to predict the assigned species of the cultures with a high degree of accuracy (0.999) as revealed by cross validation. The analogical classification used by algorithms applied in databases such as the MALDI BioTyper database enabled protein signal consistency to be incorporated into the calculations in order to increase reproducibility (Maier et al., 2006). Indeed, Mellmann et al. (2009) and Westblade et al. (2015) reported very high reproducibility levels for species designation when MALDI-TOF MS reference-based classification was used.

Biological variations in the bacterial mass fingerprint have been insufficiently studied when all samples are subject to the same cultivation, time and sample preparation conditions. However, Arnold et al. (1999) found that the age of a culture significantly influences the protein profile in the mass spectra of *E. coli* strain K-12. The presence and intensity of different peaks were observed to vary during an 84-h cultivation experiment. Interestingly, the 22–30 h cultivation time frame, corresponding to a middle stationary growth phase of *E. coli*, was found to be unstable in terms of protein expression. Significant changes were detected within subsequent 2-h time windows. Such protein variations over time are regarded as organism-specific, indicating that the uniform cultivation time prior to sample preparation for MALDI-TOF MS analysis could have an unfavorable impact. The application of a protein extraction step in sample preparation has been reported to affect the quality of mass spectra to some degree. A positive effect was mainly found in analyses of Gram-positive bacteria (Dai et al., 1999; Alatoom et al., 2011; Schulthess et al., 2013). However, the relationship between protein extraction and direct transfer in terms of biological reproducibility or mass signal stability is not discussed in any of the studies mentioned. In addition, various extraction protocols have been found to prolong sample preparation time which is noticeable when analyzing several hundred isolates.

## Conclusion

Our study highlights the limitations of MALDI-TOF MS whole-cell analysis when used for bacterial classification and identification of environmental isolates at the species level due to the lack of references in available databases. When used to dereplicate recurrent bacterial isolates, similarity-based analysis is preferable; we demonstrate that this method leads to a significant reduction in recurrent isolates, with only slightly lower precision reported as compared to the 16S rRNA gene-based approaches. It is noteworthy that the presented cosine similarity thresholds should be applied with care as they were derived from a limited sample of 49 cultures. However, our data indicate that the optimal threshold definition is primarily influenced by the biological reproducibility. Therefore, approaches that lead to high whole-cell MALDI-TOF mass spectra generation reproducibility need to be developed/established before refining the optimal threshold any further. Taking into account time and cost considerations, we concluded that MALDI-TOF MS can successfully rival the 16S rRNA gene approach in terms of high-throughput bacterial isolate binning. MALDI-TOF MS analysis also has further improvement potential unlike 16S rRNA gene analysis, whose methodological limits have plateaued. Thus, the relationship between whole-cell mass spectra and the average nucleotide identity of orthologous genes, as well as biological reproducibility issues need to be addressed in the future in order to maximize the benefits of similarity-based and reference-free approaches.

## Author contributions

Experimental design: MS and OU. Performed the experiments: MS, TS, and PJ. Analyzed the data: MS and TS. Wrote the paper: MS and OU.

### Conflict of interest statement

The authors declare that the research was conducted in the absence of any commercial or financial relationships that could be construed as a potential conflict of interest.
