# Supplementary material for: Whole-Cell MALDI-TOF MS Versus 16S rRNA Gene Analysis for Identification and Dereplication of Recurrent Bacterial Isolates
Source: Front Microbiol. 2018 Jun 19;9:1294. doi: 10.3389/fmicb.2018.01294 (PMC6018384; doi:10.3389/fmicb.2018.01294)
Supplement: Supplementary file 1 [file Image_1.PDF]

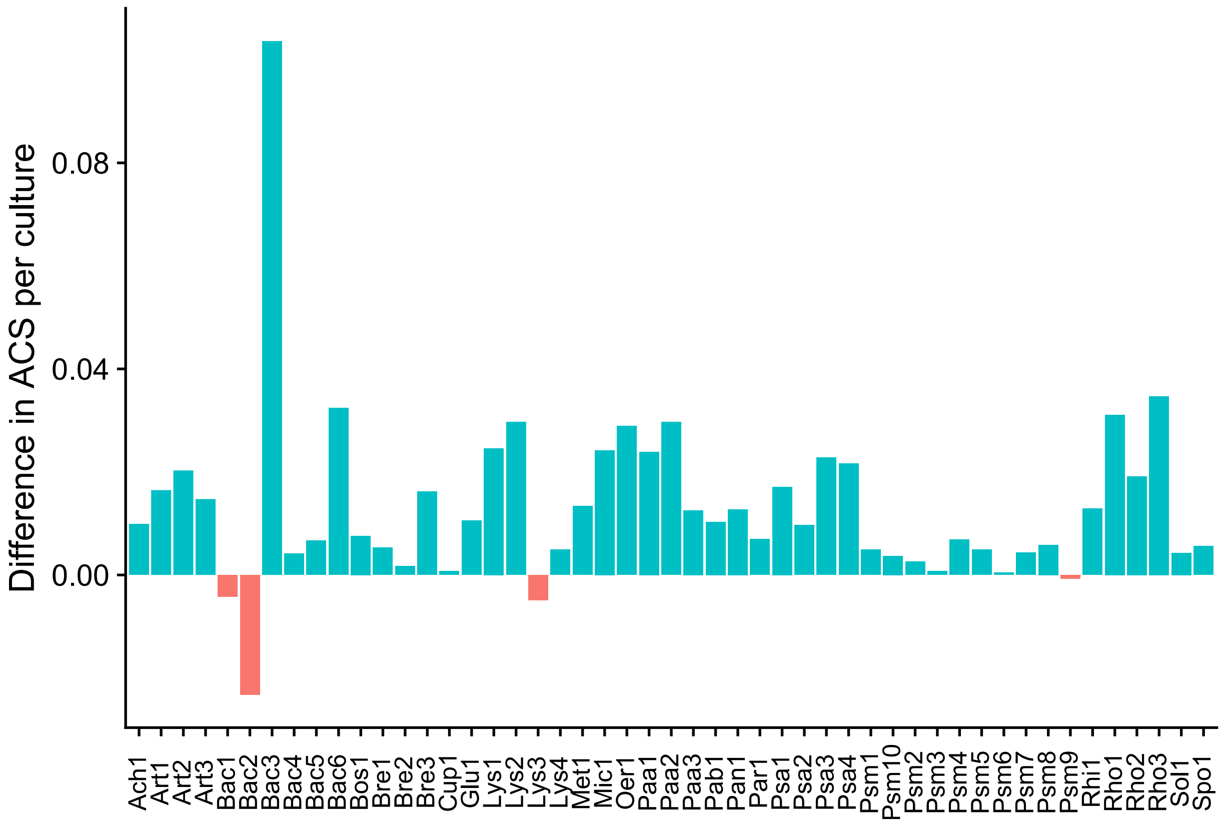

Supplementary Figure 1: The effect of mass range restriction. The bars represent a change in average cosine similarity (ACS) calculated between all mass spectra belonging to individual cultures. *Blue bars* represents a positive change, i.e. ACS is higher after the 4–10 kDa restriction compared to the full 2–20 kDa mass range whereas *red bars* represent a worse ACS per culture after the mass range restriction.
