# Supplementary material for: Whole-Cell MALDI-TOF MS Versus 16S rRNA Gene Analysis for Identification and Dereplication of Recurrent Bacterial Isolates
Source: Front Microbiol. 2018 Jun 19;9:1294. doi: 10.3389/fmicb.2018.01294 (PMC6018384; doi:10.3389/fmicb.2018.01294)
Supplement: Supplementary file 2 [file Image_2.PDF]

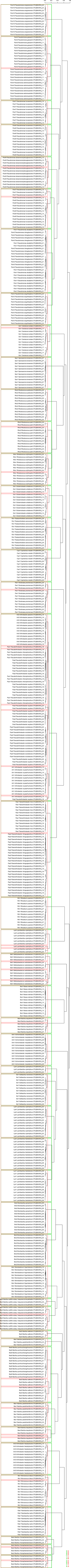

Supplementary Figure 2: UPGMA dendrogram of full mass spectra dataset (585 mass spectra). Each label consists of culture abbreviation (see Tab. 1); Genus and Species assignments by EzBioCloud Identify service (phylotype); and membership of numbered $OTUs_{[98.65\%]}$ that were constructed by UPGMA method with 98.65% 16S rRNA gene sequence similarity cutoff. *Green rectangles* – clusters defined by 0.79 cosine similarity that are analogous to $OTUs_{[98.65\%]}$; *Red rectangles* – clusters defined by 0.92 cosine similarity that are analogous to phylotypes.
